# Modified Nipple Correction Surgery Combined With Debridement for Refractory Nonpuerperal Mastitis: A Clinical Study

**DOI:** 10.1155/tbj/8818284

**Published:** 2026-07-26

**Authors:** Bing Wang, Jiachen Xu, Yue Zhou, Meina Ye

**Affiliations:** ^1^ Department of Breast Surgery, Longhua Hospital, Shanghai University of Traditional Chinese Medicine, Shanghai 200032, China, shutcm.edu.cn

**Keywords:** breast mammaplasty, case series, refractory plasma cell mastitis

## Abstract

**Objective:**

In refractory nonpuerperal mastitis, the treatment for fistulas requires surgeries or techniques that not only remove the lesion but also correct the inverted nipple. To improve the stability of nipple correction, Dr. Ye Meina modified the conventional technique and combined it with the fistula incision and debridement surgery to treat fistulas in refractory plasma cell mastitis (PCM).

**Methods:**

This study evaluated 12 female patients with a refractory history of PCM. All patients received fistula incision and debridement combined with modified nipple correction surgery. Clinical data, including patient information, treatment effects, adverse effects, and breast appearance, were collected, and all directional information has been de‐identified.

**Results:**

Follow‐up was 8–39 months. The average age of all participants was 37.1 ± 7.45 years old. All patients accepted this surgery, and postoperative pathology matched the manifestation of acute or chronic PCM. The cure rate and effective rate of these patients both reached 100% (12/12), and the recurrence rate was 0% (0/12). One patient (8.3%) encountered nipple inversion recurrence. For adverse events, two reported scar formation, and one reported reversible abnormal nipple sensation.

**Conclusion:**

The fistula incision and debridement combined with modified nipple correction could treat refractory PCM effectively by facilitating the suture removal, reducing postoperative discomfort for patients, and making smaller surgical incisions possible. Consequently, this modified surgery has shown preliminary favorable results and warrants further investigation to determine generalizability.

## 1. Introduction

Nonpuerperal mastitis (NPM), primarily including plasma cell mastitis (PCM) and granulomatous lobular mastitis (GLM), represents a group of benign, chronic inflammatory breast diseases. PCM is a chronic NPM that is commonly recognized as a recurrent and persistent disease. PCM is pathologically characterized by mammary duct expansion, plasma infiltration, and granuloma formation, and features breast or periareolar lumps and abscesses, increased comedo‐like nipple secretion, and crater nipples [1]. Although distinct in histopathology, PCM and GLM share similar clinical challenges: recurrent abscesses, complex fistulas, and nipple inversion [2]. When these conditions persist or recur despite standard treatment, they are clinically defined as refractory nonpuerperal mastitis (RNPM). In this study, we focused on the surgical management of this difficult‐to‐treat condition, regardless of the specific histological subtype.

One of the most significant causes of PCM is nipple inversion, or nipple retraction. Normal nipples stand over the areolar skin, while the retracted ones lie below the plane of the areola. Traditionally, the assessment of inverted nipples adopted the classification created by Han and Hong: Grade I retraction refers to inverted nipples that could be pulled out by hands and remain its projection without traction; Grade II retraction refers to nipples could be pulled out with difficulties and fibrosis causing retraction could be found under the nipples; Grade III retraction refers to a severe inversion that need more than manual pulling‐out, resulting from more fibrosis beneath the nipple while less support tissue under the nipple [3]. Epidemiological data on nipple inversion are sparse: In 1999, HSPark reported the prevalence of nipple inversion was 3.26% in Korean women [4], while Liu reported the rate in lactating women at 8.29% [5]; in 2012, Sapountzis and Bracaglia found the morbidity reached 10% [6]. For NPM patients, a meta‐analysis revealed that the risk of illness is 4.290 times higher than that of normal women [7].

Among all lesions, fistulas form in a relatively long period, and surgeries are highly recommended for treatment [8]. Despite the difficulties in treatment, traditional Chinese medicine (TCM) has a long history of treating RPCM and is recommended in China as the first choice because of the low recurrence rate and better preservation of the mammary function [9, 10]. In terms of RPCM with nipple retraction, it has been researched to show definite efficacy that fistula incision and nipple retraction should be both adopted based on herbal medications [11, 12]. In recent years, Dr. Ye MeiNa, the sixth‐generation successor of Gu’s TCM academic school, modified the original surgical method of nipple reconstruction. Modified nipple correction surgery may be associated with shorter recovery time and a higher rate of nipple reconstruction success. This study reports and analyzes 12 cases operated on using the new surgical method.

### 1.1. History and Different Methods of TCM in Treating Nipple Inversion

As nipple inversion is a common cause of NPM, surgeries correcting nipple inversion have been adopted to prevent recurrence. In the past 6 decades, the nipple correction for NPM patients has evolved and been refined within clinical practices. This part introduces Gu’s TCM academic school’s dedication to developing nipple correction surgeries, the common pattern of nipple correction surgery for now, and the modified nipple correction surgery developed by Dr. Ye.

### 1.2. A Brief History of Nipple Correction in NPM by Gu’s TCM Academic School

**FIGURE 1:**
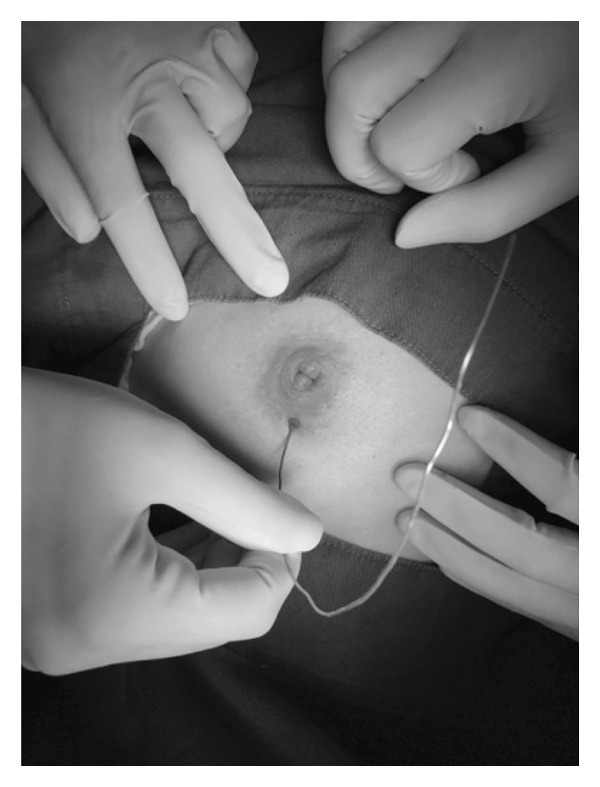
(left): A real case where the *QiuTouYinSi (a silver probe with a bulbous tip)* is applied in contemporary surgery. This patient had a fistula starting from the margin of the areola below the nipple. The fistula connected with the dominant duct, so that the other opening was in the nipple. By inserting the ball‐tip side of *QiuTouYinSi* into the opening on the skin surface, surgeons were able to follow the fistula and found the cavity inside the subcutaneous layer.

**FIGURE 2:**
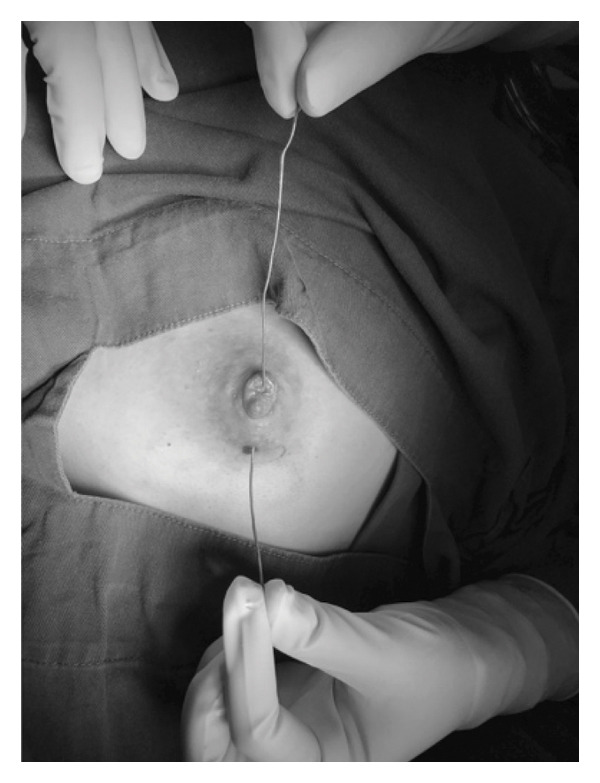
(right): A real case where the *QiuTouYinSi* is applied in contemporary surgery. By exploring the fistula with *QiuTouYinSi*, the ball‐tip side of the QiuTouYinSi could found its way out in the nipple. With the help of *QiuTouYinSi*, surgeons had a general picture of how the fistula extended, based on which the incision was made, and the diseased tissues would be under direct vision during the surgery.

In November 1954, Dr. Gu BoHua, the third‐generation successor of Gu’s TCM academic school, first treated a patient with a single breast fistula. Dr. Gu applied the thread hanging method, which was derived from treating anorectal diseases, in treating breast fistulas. By adopting *QiuTouYinSi*, a TCM surgical equipment wire made of silver with a ball tip that is flexible enough to follow fistulas (Figures 1–2) [13], Dr. Gu managed to explore the direction and openings of the fistula. He then attached threads to one end of *QiuTouYinSi* and dragged it throughout the fistula to leave the thread inside. By tightening the thread at a regular interval, one side of the fistula will be cut open to reveal the fistula as a hole. His thread‐dragging technique was an innovation at that time and was published in the *Shanghai Journal of Traditional Chinese Medicine* in 1958 [14].

In later clinical practices, Dr. Gu modified this method by replacing the thread with an elastic band because of its consistent traction. The traction freed the patients from frequent visits to the doctors for thread tightening, excessive bleeding, and long treatment time. Dr. Lu DeMing, successor of Dr. Gu, collated similar cases treated by his teacher to innovate new techniques. They also tried the surgeries: in clinical research involving 30 patients with chronic recurrent areola exudation accompanied by nipple retraction, Dr. Lu demonstrated that surgeries outperformed thread‐dragging techniques in terms of recovery time and levels of pain. He explained that the surgery was supposed to expose the fistula thoroughly and remove the necrotic tissues by cutting the nipples open, or papillotomy. Afterward, a daily dressing should be performed to promote new tissue growth and recovery [15].

The papillotomy did improve the efficacy; however, some patients’ nipples did not heal well, which affected the appearance. Dr. Tang HanJun, another successor of Dr. Gu, came up with two solutions: either replaced the surgery with thread‐dragging method (another TCM external technique: (1) dragged the thread throughout the fistula to leave the thread inside; (2) attaching external medicine on the outer part of thread before dragging the thread to allow the medication contacting the fistula surface; (3) removing the thread once the necrotic tissues were cleaned up), or performed the nipple correction after the papillotomy, which made Dr. Tang HanJun the first doctor who introduced the nipple correction in NPM treatment. He performed the surgeries in the following steps: (1) cut the nipples open; (2) loosen muscle fiber strips causing the nipple retraction until the nipples stop retracting; (3) cut the excessive skin and make interrupted sutures to close the nipple; (4) remove the stitches 5–7 days after the surgery to ensure that the nipples do not retract. Dr. Tang’s innovation significantly lowered the recurrence of NPM and was gradually adopted by other doctors [16].

### 1.3. History and Introduction of Fistula Incision and Debridement Combined With the Traditional Nipple Correction

**FIGURE 3:**
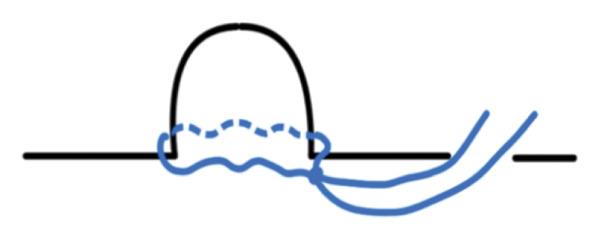
(left): A coronal sketch of the ideal position of the purse suture within the corrected nipple. The black curve refers to the nipple, and the black line represents the skin. The space between the two black lines stands for the incision. Blue curves are the general depiction of the suture, in which the dotted line represents the sutures that were placed on the distant side of the nipple, opposite to the solid line. The blue dot refers to the ideal position of the knot. As is depicted in the sketch, the knot is placed near the incision at the base of the nipple. The suture is long enough to stretch outside the incision for later removal.

**FIGURE 4:**
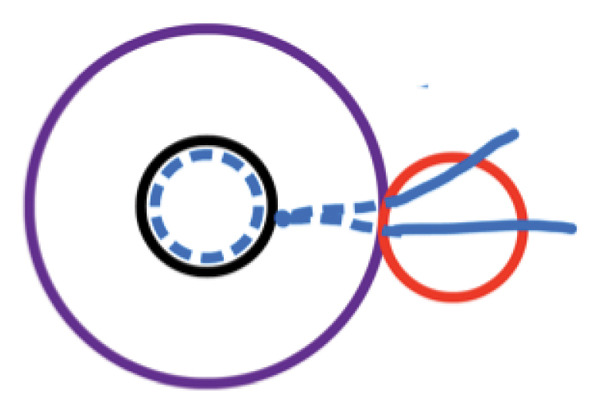
(right): A top view sketch of the corrected nipple. The black circle refers to the nipple and the concentric purple circle represents the areola. The tangential red circle stands for the incision. The blue curves, like Figure 3, are the general depict of the suture, in which the dotted line represents the sutures placed subcutaneously, opposite to the solid line. As is demonstrated in the sketch, the knot is placed just below the nipple in the cavity. Horizontally, the knot is nearly on the margin of nipple and is close to the incision, which provide convenience for the stitch removal operation.

**FIGURE 5:**
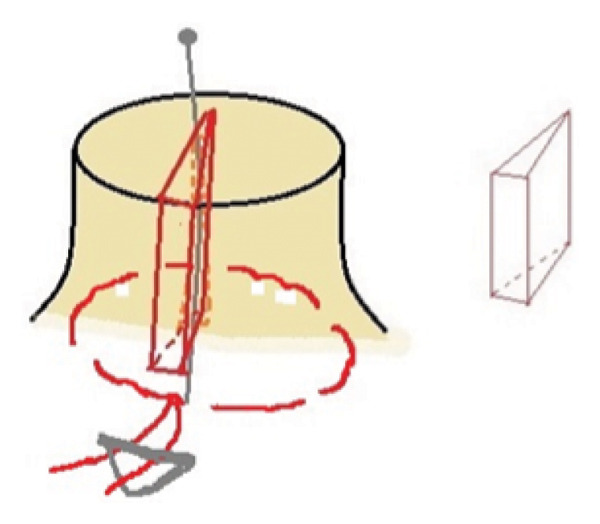
(left): A three‐dimensional sketch of the ideal position of *QiuTouYinSi* in the diseased nipple. The yellow part refers to the nipple; the red circle sketches the cavity under the nipple; the grey line with a ball‐like ending is *QiuTouYinSi*; the red triangle prism demonstrates the tissues needed to be cut; the grey triangle below shows the incision for the surgery. Once the incision (gray triangle) is made, surgeons can explore how the fistula stretches by putting the *QiuTouYinSi* inside the nipple hole. This *QiuTouYinSi* is soft enough to bend according to the shape of the fistula, so surgeons can pull it out from the incision. After the exploration process, surgeons understand where the fistula is, so that by cutting the tissues above the *QiuTouYinSi,* surgeons can open the fistula.

**FIGURE 6:**
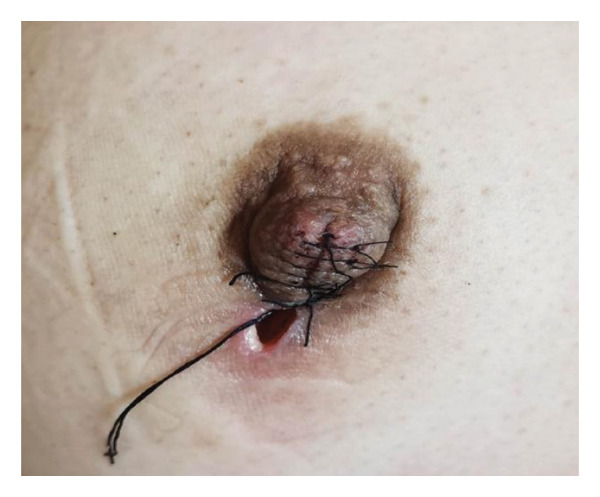
(right): A real case in which the traditional fistula incision surgery has been done. On the six o’clock of the nipple, an incision could be observed that has been made according to the direction of *QiuTouYinSi*.

With more research and clinical practice, the Gu’s as well as other doctors have improved their strategy to treat NPM. Dr. Chen HongFeng and Dr. Chen YiQin, both fifth‐generation successors, combined the abscess incision and drainage with the nipple correction. The surgical solution for the NPM aimed to remove the ulcers, masses, or abscesses within the breast and correct the nipple inversion. This surgery mainly includes eight steps: (1) patients are placed in supine position with the affected side of the upper limb abducted. The operative area is prepped and draped in a sterile fashion; (2) all patients are under total intravenous anesthesia; (3) Incisions should be designed to cover the ulcer. Preferably, the incision margin should overlap the areola. In general, the size of the incision is based on the size of the ulcer, whose ideal length ranges from 1.0 to 2.5 cm. The *QiuTouYinSi* could be employed to explore the fistula by introducing it into the nipple and pulling it out from the ulcer or the incision. Upon successful localization, tissues are dissected layer by layer along the *QiuTouYinSi*. After cutting the skin, subcutaneous tissue, mammary glands, and nipple, the cavity behind the nipple would be exposed. (4) Clean the cavity and remove the secretion and the surrounding inflammatory tissues. The specimen is supposed to be sent to the Department of Pathology for further examination; (5) Separate the nipple and its subareolar tissues to loosen the milk ducts and muscle fiber strips causing the nipple retraction. Pull out the nipple completely for reconstruction. (6) Put a purse‐string closure on the base of the new‐shaped nipple and make sure the closure is not too loose or too tight. The knot should be placed at the base of the nipple with the shortest distance to the incision. The remaining part of the suture should be long enough to extend outside the incision (Figures 3–6). (7) Close the nipple with interrupted stitches. (8) Complete the hemostasis process before rinsing the cavity with warm saline. Drain out all the blood and other fluids and suture the cavity layer by layer.

The sutures are kept tight before their removal. To remove the stitch, surgeons tend to pull the suture straight and explore into the cavity to find the exact position of the knot. In cases with a limited cavity, some knots could not be seen directly, which made the removal process difficult.

### 1.4. Introduction of Fistula Incision and Debridement Combined With Modified Nipple Correction

The former surgery pattern has proven its efficacy by different experts [12, 13, 17, 18]. However, there is still room for improvement: (1) The process of suture removal is done in a relatively limited cavity. Sometimes surgeons’ views are limited during this invasive manipulation. If the incision is too far from the knot (> 2.5 cm), surgeons would face more challenges and sometimes cause bleeding or collateral repair of the partial tissues. (2) The growth rate of granulation tissue may influence the best timing of the suture removal. For those whose granulation tissue grows fast, the knot might be covered within the tissues, increasing the difficulty of suture removal. (3) As with all the other nipple correction surgeries based on purse‐string sutures, the suture tension determines the efficacy of correction. Complications like papillary necrosis happen with high‐tension sutures, while nipple correction may fail in cases with low‐tension sutures. Therefore, the volume of the cavity, the distance between the incision and the suture knot, and the suture tension are the determinants of traditional nipple correction surgery.

**FIGURE 7:**
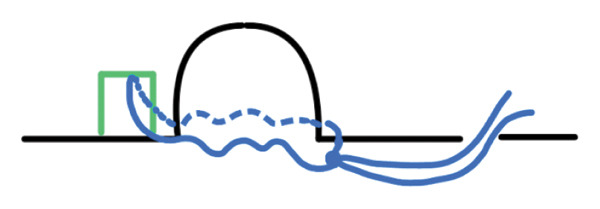
(left): A coronal sketch of the ideal position of the modified corrected nipple. The green rectangle indicates the gauze. The black curve refers to the nipple and the skin, in which the spaces stand for the incision. Blue curves demonstrate the suture. The blue dot refers to the ideal position of the knot. As is depicted in the sketch, the purse‐string suture comes out of the nipple on the opposite part of the nipple from the incision. The sutures fix the gauze and pass again into the nipple. The exposed part of the suture provides a special location for surgeons to adjust the tension of the suture or remove the suture once the correction is done.

**FIGURE 8:**
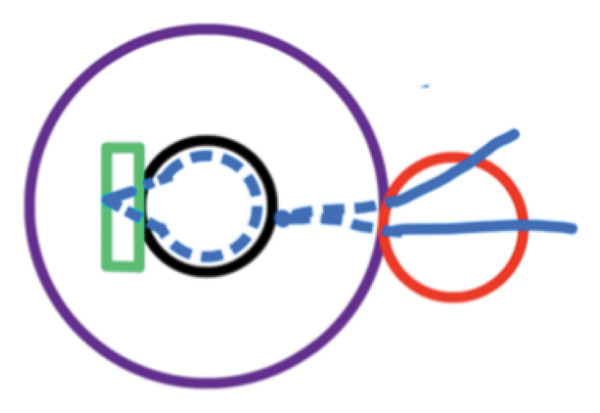
(right): A top view sketch of the corrected nipple. The green rectangle stands for the gauze and other annotations remain the same as Figure 8. As is demonstrated in the sketch, the gauze, the nipple and the incision are on the same line with the nipple in the middle. This sequence ensures the best stability of the gauze, or it might slip when encountered strike or excessive movements.

**FIGURE 9:**
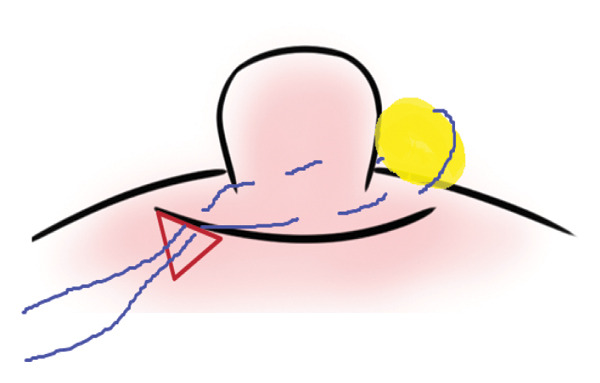
(left): A three‐dimensional sketch of the ideal purse‐string suture fixed with the help of gauze. The black lines represent the margin of the areola, nipple, and skin; the blue dotted lines refer to the surgical suture; the red triangle is the margin of the surgical incision; on the opposite part of the nipple, the yellow shape displays the position of the gaze. In this scenario, the suture enters the cavity through the incision to make a purse‐string suture on the wall of the cavity. When the suture proceeds to the opposite wall, it should traverse the nipple. Before the suture goes back into the cavity, a folded gauze should be placed between the suture and nipple to keep both the suture and nipple stable. After the remaining purse‐string suture is finished, the rest of the suture comes out from the incision.

**FIGURE 10:**
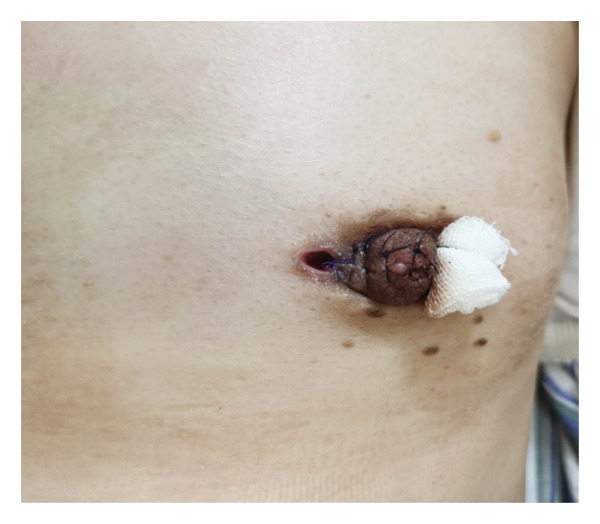
(right): A real case of receiving modified nipple correction surgery. In this case, the incision was close to the nipple because of the limited areola. The purple suture used for a purse‐string suture immobilizes the gauze. Both the ends of the suture came out of the incision.

To tackle these problems, Dr. Ye MeiNa made a modification based on the original surgical method of nipple reconstruction. Most of the procedures are similar and are also presented in eight steps. The anesthesia, incision design, exploration of the fistula with QiuTouYinSi, and the debridement of affected tissues are similar. In the nipple correction process, put a purse‐string closure on the base of the new‐shaped nipple and make sure the closure is not too loose or too tight. Compared with the traditional purse‐string closure, the improved closure starts at the base of the nipple on the side of the incision next to the areola, and the suture goes through the skin on the opposite side of the nipple, and a piece of gauze of 1 × 0.8 cm in size is stitched. The gauze is thus fixed beside the nipple on the opposite side of the incision. After fixation, pass the suture line through the skin again into the subareolar sore cavity to complete the second half of the purse‐string closure (Figures 7–10). After the closure, the hemostasis process.

This seemingly slight modification has shown a feasibility signal in stability and flexibility: with an exposed segment of suture in the air, surgeons would not have difficulty in finding the knot. They would rather cut the suture and pull the remaining part extending from the incision to remove the sutures safely. No exploration or detection inside the cavity is needed so that unexpected complications like bleeding are avoided. Also, the additional suture fixing the gauze leaves space for surgeons to readjust the tension. The judgement of tension relies on surgeons’ experience to a large extent. Overtightened sutures could be loosened by replacing them with smaller gauze and dragging the sutures to maintain the optimal tension, while the loose ones could be frapped again with the help of this additional segment of suture. In short, the volume of the cavity, the distance between the incision and suture knot, and the suture tension are no longer the determinants of this surgery. This modification ensured a safer suture‐removal process and a second chance to adjust the suture tension.

To investigate the technical feasibility and initial safety outcomes, this research aims to report a modified nipple correction surgery employed in NPM treatment. This modified surgery appeared feasible and showed encouraging short‐term outcomes.

## 2. Material and Methods

### 2.1. Clinical Data

Twelve patients diagnosed with RPCM whose areolar lesions remained unhealed and relapsed frequently from January 2023 to August 2025 were included in this study. Patients completed a follow‐up data collection with a deadline of April 2026. Patients’ written informed consent has been obtained for the use of their de‐identified medical information for academic publication and teaching purposes.

### 2.2. Clinical Manifestation

Patients involved in this research may have symptoms like (1) areolar lumps with reddish discoloration and forming pus around 7–10 days; (2) pus with stink smell and refractory or repetitive lesions; (3) medical history of nipple retraction with comedo‐like nipple secretion; (4) affected breast with nipple retraction; (5) refractory ulcerations resulting in fistulas which could be traced with the help of *QiuTouYinSi*.

### 2.3. Inclusion and Exclusion Criteria

Patients who satisfied the following criteria were included: (1) clinical symptoms match the diagnostic criteria of PCM [19, 20]; (2) imaging examination (e.g., ultrasound, mammography, and magnetic resonance imaging) or pathological test suggests PCM and is confirmed with postoperative pathology. The RPCM patients share the medical history of refractories and nipple retraction.

The exclusion criteria could unfold as follows: (1) patients in the breastfeeding period who suffer acute mastitis; (2) breast tuberculosis and other specific breast abscesses; (3) patients with other malignant diseases or other severe diseases affecting incision healing, including cardiac or cerebrovascular diseases, severe respiratory, digestive, hematological, urinary, and neurological diseases.

### 2.4. Treatment

#### 2.4.1. Preoperative Treatment

Patients would take the internal traditional Chinese herbal therapies before the operation. The Chaihu Liver Cleansing Paste was employed in this study, which consist of 6 g of *Bupleurum*, 9 g of *Scutellaria baicalensis*, 9 g of *Radix Curcumae,* 15 g of *Atractylodes macrocephala*, 15 g of *hawthorn*, 15 g of *Poria*, 15 g of *Salvia Miltiorrhiza*, and 30 g of *Taraxacum officinale* per dose and can be adjusted no more than 5 herbals according to patients’ conditions.

#### 2.4.2. Surgical Procedure

The fistula incision and debridement combined with modified nipple correction surgery can be performed in the following seven steps: (1) patients are placed in a supine position with the affected side of the upper limb abducted. The operative area is prepped and draped in a sterile fashion.; (2) all patients are under total intravenous anesthesia; (3) Incisions should be designed to cover the ulcer. Preferably, the incision margin should overlap the areola. In general, the size of the incision is based on the size of the ulcer, whose ideal length ranges from 1.0 to 2.5 cm. The *QiuTouYinSi* could be employed to explore the fistula by introducing it in the nipple and pulling it out from the ulcer. Upon successful localization, tissues are dissected layer by layer along the *QiuTouYinSi*. After cutting the skin, subcutaneous tissue, mammary glands, and nipple, the cavity behind the nipple would be exposed (Figures 3 and 4). (4) Clean the cavity and remove the secretion and the surrounding inflammatory tissues. The specimen is supposed to be sent to the Department of Pathology for further examination; (5) Separate the nipple and its subareolar tissues to loosen the milk ducts and muscle fiber strips causing the nipple retraction. Pull out the nipple completely for reconstruction. (6) Put a purse‐string closure on the base of the new‐shaped nipple. The closure starts at the base of the nipple on the side of the incision next to the areola, and the suture goes through the skin on the opposite side of the nipple, and a piece of gauze of 1 × 0.8 cm in size is stitched. The gauze is thus fixed beside the nipple on the opposite side of the incision. After fixation, pass the suture line through the skin again into the subareolar sore cavity to complete the second half of the purse‐string closure. (7) Complete the hemostasis process before rinsing the cavity with warm saline. Drain out all the blood and other fluids and suture the cavity layer by layer.

#### 2.4.3. Postoperative Treatment

After the operation, the affected breast should be wrapped up under pressure with a multihead axillary thoracic belt. A daily dressing change is needed for 2–3 weeks before the stitches are removed.

### 2.5. Observation Indexes and Efficacy Evaluation

To observe the efficacy of fistula incision and debridement combined with modified nipple correction in treating RPCM, four indices should be included that consist of the average postoperative wound healing time, the healing time, the recurrence rate, the adverse reactions, and patients’ satisfaction.

#### 2.5.1. Efficacy Evaluation

Record the symptom and sign scores before herbal medication, before surgery, and after the overall treatment. The symptom and sign scores were established according to the *Technical Guideline for the Preparation Research of Clinical Trial Drugs of New Chinese Medicine (Trial)* [21, 22]. The symptoms and signs could be scored as 0, 1, 2, or 3 based on the severity. Furthermore, the efficacy was determined as cured, significant, effective, and ineffective on the basis of the magnitude of change on patients’ symptom and signs with a reference to *Criteria of diagnosis and therapeutic effect of internal diseases and syndromes in TCM*: (1) cured: Patients with ≥ 95% resolution of symptoms and signs, complete resolution of lumps, and healed fistulas without any other systemic symptoms; (2) significant: Patients with ≥ 70% resolution of symptoms and signs, lumps shrink, fistulas nearly healed; (3) effective: Patients with ≥ 30% resolution of symptoms and signs, less reddish skin and pain, fistula partly healed; (4) ineffective: no significant resolution of symptoms and signs could be observed, lumps and fistulas remained or even swell and become purulent. To be specific, those who lump reappears in the same location with or without redness, swelling, and pain, or pus comes out from ulceration 3 months or more after the cure, were defined as refractory patients. Meanwhile, in this study, the researchers define the overall effective rate as (number of cured patients + number of patients with significant effect + number of effective patients)/total number of patients × 100%.

#### 2.5.2. Evaluation of Breast Aesthetics

The evaluation of breast aesthetics could be classified into four categories: (1) Excellent: no significant difference in breast size and shape from the contralateral breast, normal appearance; (2) Good: retraction of the nipple and/or skin changes (scarring) remaining less than 1/4 of the original; (3) Fair: retraction of the nipple and/or skin changes (scarring) involving 1/2 to 1/4; 4) Poor: retraction of the breast and/or skin changes (scarring) affecting more than 1/2 of the breast.

#### 2.5.3. Postoperative Follow‐Up

All patients have made a follow‐up visit postoperatively in our outpatient clinic. Another online or telephone follow‐up will be done in April 2026.

#### 2.5.4. Intervention Adherence and Tolerance

Outpatient follow‐up records and online follow‐ups are the main basis for adherence, and tolerance is based on monitoring and recording all adverse events throughout the study period by patients and physicians. The severity of adverse events is classified according to the Common Adverse Event Evaluation Criteria (CTCAE) Version 5.0 of the National Cancer Institute of the United States.

## 3. Results

### 3.1. General Data

All the patients involved were female. Their age ranged from 26 to 55 years old and averaged (37.08 ± 7.45) years. The course of the disease lasted (9.25 ± 8.91) months. All the patients were recorded with a repetitive history of PCM or GLM, in which the longest course recorded was 42 months. Seven patients had received a surgery before this research, in which five of them accepted surgeries in another hospital, while two patients underwent biopsy. No patients reported smoking or alcohol consumption history before. One patient suffered from GLM with nipple inversion in both breasts, so she underwent the surgery on both sides (Table 1).

**TABLE 1:** General clinical data of the patients.

Items	Patients involved
Age (years, *x* ± s)	37.08 ± 7.45
Maximum radiological parameter of lesions (mm, *x* ± s)	25.4 ± 16.5
Number of lesions	
1–2	2
3–4	3
≥ 5	7
Distribution of lesions (left/right)	11/2
Enlargement of lymph nodes (cases)	8
Patients with surgical history (cases)	7
Surgical history for PCM or GLM (times, *x* ± s)	0.75 ± 0.92
Courses of disease (months, *x* ± s)	9.25 ± 8.91
Childbirth history (times, *x* ± s)	1.16 ± 0.37
Smoking history (cases)	0
Alcohol consumption history (cases)	0
White blood cell count (^∗^10^9^, *x* ± s)	6.45 ± 1.66
Neutrophil percentage (%, *x* ± s)	59.5 ± 18.98
C‐reactive protein (cases, mg/L)	
< 0.50	5
≥ 0.50	7

### 3.2. Efficacy Analysis

All 12 cases in this study were cured; the overall effective rate of treatment was 100% (12/12), the cure rate was 100% (12/12), and the recurrence rate was 0% (0/12); the average healing time was 18.25 days (ranging from 7 days ∼ 31 days). No allergies or adverse reactions to traditional Chinese medications were reported in this study.

### 3.3. Assessment of Nipple Shape and Overall Satisfaction

In this study, 11 patients’ postoperative nipple shapes were assessed as excellent, and 1 was good. Ten patients were satisfied with the postoperative breast shape, and the other two marked “relatively satisfied”. (Table 2).

**TABLE 2:** Therapeutic efficacy of patients.

Items	Patients involved
Length of hospital days (days, *x* ± s)	18.25 ± 5.66
Corrected nipple height (mm, *x* ± s)	8.00 ± 3.91
Corrected nipple parameter (mm, *x* ± s)	10.75 ± 1.54
Efficacy of surgery	
Cure	12
Significant	0
Effective	0
Ineffective	0
Evaluation of breast aesthetics	
Excellent	11
Good	1
Fair	0
Poor	0
Recurrence (cases)	0

### 3.4. Adverse Event

Common adverse events in nipple correction surgery include poor blood circulation in the areola area, abnormal nipple sensation, scar formation, incision pain, fat liquefaction, contact dermatitis, infection, and reduced lactation. Among 12 patients, one patient reported a numb sensation in the affected nipple. However, the abnormal sensation faded 6 months after surgery. Two patients reported that the incision formed a scar after the surgery. No other adverse events were reported.

## 4. Case Report

A female, 31 years old, complained of a right breast lump that had been ulcerated and purulent repeatedly for 16 months. Initially, the patients found a right areolar lump that was red, swollen, and hard to touch. The patients did not undergo systematic treatment. Nine months later, her right areola was red, swollen, and purulent again. After being treated with antibiotics and Chinese herbs, the patient reported that the affected area improved, and the ulceration healed. After another 7 months, the lumps reappeared on the original site. The breast ultrasound from another hospital showed inflammatory lesions in the right breast and no enlarged axillary lymph nodes.

Physical examination: Bilateral nipples retracted. A firm, irregularly shaped lump (approximately 3 × 4 cm) was found posterior to the right areola with indistinct borders. Another 0.2 cm × 0.3 cm ulcerated lesion with crusting was visible above the right areola. TCM diagnostic information: red tongue with thin white coating; thin and wiry pulse.

**FIGURE 11:**
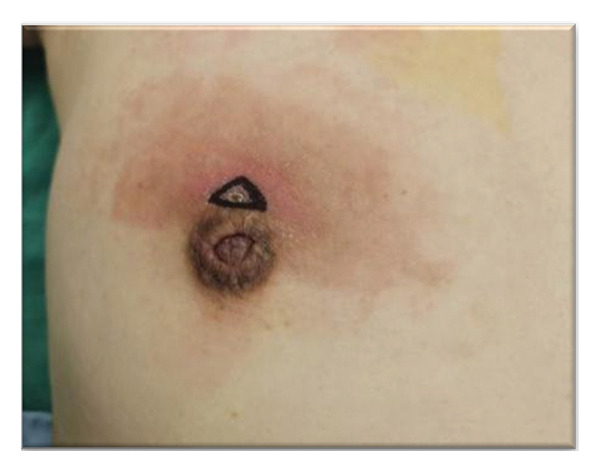
Preoperative image of the affected breast. The skin above the nipple was red, and an ulceration lesion could be observed on the margin of the areola. The designed surgical incision was marked with a black marker.

**FIGURE 12:**
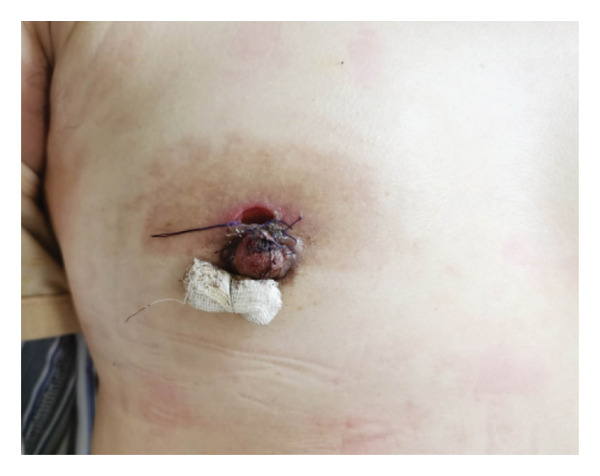
Postoperative image of the right breast (Day 1). The triangle incision was close to the nipple, and a piece of gauze of 1 × 0.8 cm in size was stitched on the opposite side to keep the nipple pulled out.

**FIGURE 13:**
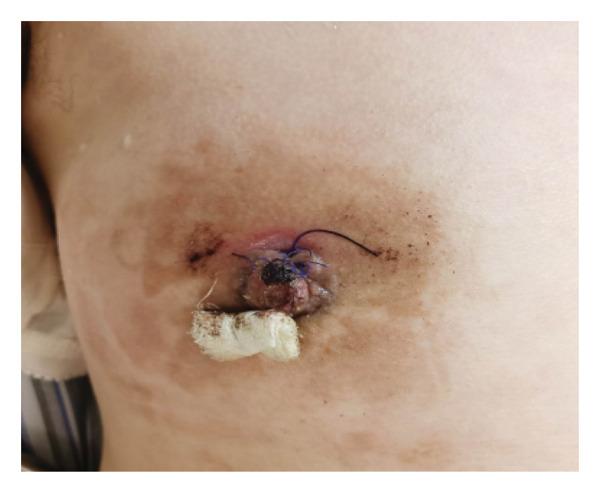
Postoperative image of the right breast (Day 10). The incision healed while the sutures remained to maintain the reconstruction effect. The gauze had been effectively sustaining the ideal position of the affected nipple.

**FIGURE 14:**
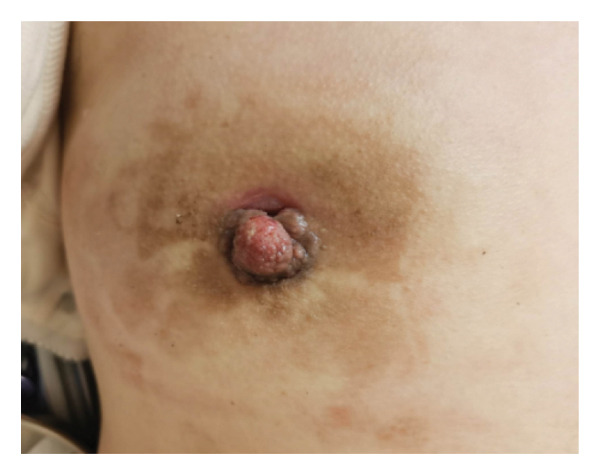
Postoperative image of the right breast (Day 14). The sutures were removed. Pigmentation was still present at the lesion site. However, the incision was concealed inside the areolar so that it was nearly invisible. The nipple’s appearance significantly improved.

This patient was diagnosed with PCM and accepted surgery. Postoperative physical examination: The right nipple was not inverted. There was a triangular incision (approximately 10 × 8 mm) above the right nipple. This incision led to a cavity 25 mm in depth from the areola and the nipple. At the early postoperative stage (about 7 days), gauze strips covered with *Hongyou Ointment*, a traditional Chinese ointment that drains out pus and removes the putridity, were employed to pack the cavity to remove the necrotic tissues. Once the necrotic tissues were removed and the wound started to heal, the dressing procedure would require flushing the wound cavity with saline and packing the cavity with gauze strips soaked with *Kangfuxin Liquid,* a widely used solution that promotes blood circulation, nourishes body fluids, and stimulates muscle growth [23]. The patient received 10 daily dressing changes until the wound healed. Fifteen days after the surgery, the purse‐string suture was removed (Figures 11–14).

In this case, our team adopted fistula incision and debridement combined with modified nipple correction to deal with an areolar fistula. The fistula was localized with the help of *QiuTouYinSi* and eliminated through a small incision. Meanwhile, the nipple was reconstructed and immobilized at an ideal position. The daily dressing changes further consolidated the surgical results. The patient recovered in 17 days, and no recurrence or nipple re‐inversion has been reported to date (over 1 year). The patient has been satisfied with the surgery.

## 5. Discussions

As a feasibility and technical report for a new surgical method, this research, unlike a superiority trial, did not establish a concurrent control group. Therefore, a comparison was made with historical data from our center to reveal the feasibility.

The historical data were published in 2022, in which the traditional nipple correction surgery was applied on 150 female PCM patients with areola fistulas, and 93 patients were RPNM. According to the past research, it has been revealed that the modified nipple correction outperformed the traditional one in terms of breast aesthetics.

In Table 3, we compared our results with historical data from the same center (same surgical team, similar perioperative protocols) as a benchmark reference to provide a rough context, not as a statistical proof of superiority. The comparison is presented as exploratory and descriptive without claims of superiority.

**TABLE 3:** Comparison with historical data from our center.

Items	Historical data	Current data
Patient number (cases)	150	12
RPNM patient number (cases)	93	12
Average Age (years)	32	37.23
Course of disease (months)	22.5	9.25
Efficacy (rate, %)	100	100
Recurrence rate (rate,%)	0	0
Recurrence patient number (cases)	0	0
Inversion recurrence rate (rate,%)	13.3	8.3
Inversion recurrence patient number (cases)	20	2

### 5.1. Progress in Etiological Research of PCM

PCM, as a chronic nonlactational or nonpregnancy‐related mastitis, has a pathogenesis that remains unveiled. While PCM and GLM differ in etiology, our study suggests that for refractory cases characterized by fistulas and nipple retraction, the surgical principle remains consistent: complete removal of the inflammatory debridement combined with correction of the underlying anatomical defect (nipple inversion).

Factors associated with PCM may include nipple retraction [24–26], abnormal changes in hormonal levels (such as elevated prolactin [25, 27, 28]), immune dysfunction [29], and breast trauma, etc [30]. Recent studies also shed light on microbial infections [31, 32]. Inverted nipples can obstruct the drainage of secretions from the mammary ducts. The accumulated secretions may block the ducts, causing ductal dilation. In adverse, the ducal dilation could stimulate more ductal secretion. This vicious circle stimulates inflammatory reactions in the surrounding tissue and duct walls, leading to the formation of lumps. Ulcerations of these lumps lead to fistulas. Such conditions tend to recur frequently and are difficult to cure [33, 34]. Researchers have found proof of high relevance between nipple retraction and PCM. Wu JingJing et al. investigated potential factors associated with 270 cases of PCM, in which nipple retraction accounted for 51% [35]. Jia Ming et al. conducted an analysis on the clinical characteristics and effects of surgical interventions on PCM. In the 91 patients under research, congenital nipple retraction was found to be the primary cause of PCM, and consequently, the nipple retraction correction turned out to be an effective measure in preventing such disease [36]. In recent years, some researchers have pointed out that ductal stasis should be the initial and crucial part of the pathogenesis of PCM, with nipple retraction or inversion being the primary cause of ductal stasis. Anatomic causes like nipple retraction result in a ductal clog that inhibits drainage, encouraging the accumulation to stimulate the duct walls to proliferate inflammatory cells. The cells subsequently induce fibrous tissue proliferation, which triggers an antigenic response in the surrounding glandular tissue and ultimately leads to plasma cell infiltration and inflammatory reactions. Furthermore, PASTA V. et al. pointed out that PCM is typically unilateral, though occasionally bilateral. Clinically, it remains more common in women with nipple inversions, which explains how the anatomical factors potentially may contribute to its pathogenesis to a larger extent than other pathogenesis.

### 5.2. Current Clinical Practices of TCM or Western Medicine on PCM

Currently, the diagnosis and treatment of PCM is reliant on the experience of doctors, and thus little consensus has been reached. Anti‐inflammatory drugs or glucocorticoids could suppress inflammatory responses and alleviate the clinical symptoms. In some cases, surgical interventions should be considered as an indispensable measure to remove the diseased tissues and consequently accelerate the course of treatment. TCM has demonstrated distinct therapeutic advantages through decades of practice, so that TCM has established itself as the pioneer of PCM treatment by combining oral medications and surgeries. Based on individualized symptom differentiation, TCM formed its special therapeutic regimens for PCM patients, which have developed distinctive and effective diagnostic and therapeutic protocols. TCM provided mature methods in oral medications, external methods, and surgeries, and TCM breast surgeons have been developing solutions by merging the advantageous ones together. Researchers have found the TCM herbal formula effective in many ways. A nonrandomized controlled trial by Liu et al. investigates the efficacy of an herbal drug combination, identified using a knowledge graph approach, for treating PCM. This study involved 120 patients, divided into an intervention group receiving the novel herbal combination alongside conventional Western medicine and a control group receiving Western medicine only. The combined therapy group demonstrated a statistically significant improvement in clinical symptoms and a reduction in local inflammatory signs compared to the other group. Furthermore, the combination therapy was associated with a lower recurrence rate, which revealed that the integrated approach outperformed adopting Western medicine only [37].

Apart from herbal medications, some researchers focused on typical Chinese ointment and proved the efficacy of their combination. Zhang Ti et al. adopted QingXiao ointment on PCM patients in the mass stage. Researchers applied the ointment externally to the affected breast and gave the XiaozhongSanJie Formula (compositions: *Dipsaci Radix* 10 g, *Cervi Cornu* slices 10 g, Baked *Ginger* 5 g, *Sinapis alba* 10 g, *Cortex Cinnamomi* 10 g,vinegar‐processed *Bupleurum chinense* 10 g, *Dianthi Herba* 30 g, *Prunella Vulgaris L.* 10 g, *Astragali Radix* 10 g, *Dandelion* 15 g, *Forsythiae Fructus* 15 g, malt 30 g) for patients to take for one week. Results demonstrated that Qing Xiao ointment effectively reduced the size of breast masses, alleviated local inflammation, and improved clinical symptoms. The authors conclude that Qing Xiao ointment served as a beneficial noninvasive external therapy for managing the mass stage of PCM, potentially by promoting blood circulation, resolving stasis, and reducing inflammation. It offers a conservative treatment option for patients seeking alternatives to surgical intervention. Alongside employing the herbal medications alone, researchers innovated by combing the herbal medications with surgeries: He Liu et al. assessed the integrated treatment of refractory PCM by using the TCM formula “Jiang Ru Fang” (composition of single prescription: *Bupleurum chinense* 9 g, *Scutellariae Radix* 9 g, *Citri Reticulatae Pericarpium Viride* 9 g, *Pericarpium Citri Reticulatae* 9 g, *Bidens Bipinnata L.* 18 g, *Prunella Vulgaris L.* 30 g, *Rhizoma Curcumae* 18 g, *Radix Paeoniae Rubra* 15 g, Radix Curcumae 12 g, *Astragali Radix* 15 g, *Ostreae concha* 30 g, *Sargassum* 30 g, *Platycodon grandiflorum* 6 g, processed‐ *Glycyrrhiza* 6 g. If swelling is present, add *Lonicera japonica* 9 g and *Dandelion* 12 g. If the mass is prominent, add *Persicae Semen* 9 g) in combination with mammoplasty and continuous negative pressure irrigation technology, and 36 patients in total were involved. The total efficacy reached 94.4% with complete healing achieved in most cases [38]. The combination of TCM formulas and TCM external methods also demonstrates remarkable efficacy: Wu XueQing et al. adopted oral medications that clear the liver and disperse heat (compositions: *Bupleurum chinense*, *Polygonum cuspidatum*, *Gardeniae Fructus*, *Scutellariae Radix*, *Hedyotis Diffusa*, *Rhizoma Curcumae*, *Radix Paeoniae Rubra*, *Crataegi Fructus*, and *Fructus mume*). The total efficacy of the internal–external therapy reached 100% with a low recurrence rate of 1.82%. Therefore, this method was evaluated as clinically safe and effective [39]. Qiu Pin et al. evaluated the efficacy of pricking‐cupping bloodletting therapy on PCM during the mass‐formation stage. In a clinical observation of 93 patients, the treatment demonstrated a total effective rate of 95.7% with the effect of reduced mass size, alleviated pain, and improved local inflammation, which made the pricking‐cupping bloodletting therapy an excellent choice at the early stage of PCM [40]. Furthermore, more TCM surgeons have developed specialized TCM surgery patterns that outperform the conservative ones. This study by Tang et al. presents a traditional surgical TCM approach for treating 148 patients with PCM. The method involves surgical incision, thread‐dragging drainage (passing 4–6 strands of thread through the fistula tract with Jiuyidan applied and pulling the thread back and forth to administer the medication), and promotion of tissue regeneration (during the dressing change, applying debridement medication). Results showed the technique successfully controlled infection, drained pus, and promoted granulation tissue formation, and the recurrence rate was low [41]. Wang Bin et al. evaluated the clinical efficacy of Gu’s (Gu’s academic school, a decent TCM academic school derived from Shanghai) surgical approach, specifically minor incision debridement, for treating lactiferous fistula caused by PCM. The surgical approach could be divided into four steps: (1) Gently insert the *QiuTouYinSi* through the ulcerated opening in the affected nipple‐areola complex. Guide it along the duct until it emerges through the nipple orifice. (2) Make a radial incision along the silver wire to dissect the nipple and areola. (3) Fully expose the subcutaneous cavity within the nipple. Remove necrotic tissue from the cavity. Clear the lesion septum. (4) After removing necrotic tissue from the cavity, suture the nipple. In a retrospective analysis of 72 patients who accepted such a surgical method, the treatment achieved a 100% cure rate with a very low recurrence rate of 2.78% and a high patient satisfaction rate of 95.83%. The average wound healing time was approximately 24 days [13]. In a nutshell, TCM has an extensive history of success and offers many advantages in the treatment of PCM, and is recommended in China as the first choice alongside other methods.

### 5.3. The Fistula Incision and Debridement Combined With Modified Nipple Correction

The refractory PCM with recurrent abscesses, sinus tracts, or fistulas in the areolar region, particularly when accompanied by nipple retraction, has been presenting challenges for surgeons. In general situations, the fistula could extend from the external opening to deep layers of the areola or the nipple. Although the traditional anti‐inflammatory treatment combined with incision and drainage of the abscess or cavity‐packing with gauze strips healed the external openings, the fistulas inside remained. Once the immunities declined or secretions accumulated, the external opening became inflamed and ulcerated and discharged pus. Not only do such vicious circles increase the patient’s suffering, but they also prompt researchers to analyze the causes of recurrence during treatment. In our studies, all participants were suffering from the refractory PCM with nipple retractions and multiple symptoms, including lumps, abscesses, and fistulas.

Through years of clinical practice, our team has continuously explored and innovated, establishing a comprehensive treatment plan with a TCM internal–external combined strategy to provide patients with more effective care. First, during the acute phase of PCM, medications effectively control the progression of these symptoms, including oral herbal formulas and various ointments. The key to this phase is to “postpone” the inflammation to create favorable conditions for surgery. Second, once the redness and swelling have been alleviated, timely surgical intervention is indicated. The surgical approach employs a “duet” technique combining fistula and surrounding necrotic tissue excision with nipple reconstruction. Making an incision along the fistula tract from the lateral breast ulceration toward the nipple helps thoroughly remove lesions concealed within the nipple. The essence of such a strategy is to “eliminate” the PCM site with “minimum” incision. Third, retracted nipples are not only a common manifestation of PCM but also significantly affect patients’ life quality. Nipple correction surgery is necessary to restore the inverted nipple. Alongside improving breast aesthetics, the procedure facilitates the smooth discharge of secretions from the milk ducts, which prevents the accumulation of secretions within the ducts. Otherwise, the accumulation would stimulate the duct walls and trigger mastitis. Fourth, the dressing change is crucial to solidify the surgical effects. The incision is left open without suturing and heals gradually with the help of dressing changes. The advantage of an open incision is that it facilitates the smooth drainage of residual secretions or necrotic tissues within the cavity. During daily dressing changes, these substances are removed progressively, thus creating favorable conditions for wound healing. More importantly, the opening incision facilitates purse‐string suture removal. In 2022, our team reviewed 150 cases of refractory PCM treated with nipple incision and fistula excision. The cure rate was 100%, with no recurrence observed during the 2‐year follow‐up [30]. However, our team has noticed that some patients’ nipples retracted to different extents after the correction, jeopardizing the aesthetic appearance of the breast . To elevate the success rate of nipple reconstruction, our team analyzed all critical steps, including surgical procedures, postoperative dressing changes, and suture removal, striving to identify the causes affecting the outcome of the reconstruction. On one hand, the malformation of nipples (e.g., short nipple‐areola complex) inhibits the restoration of nipple appearance. On the other hand, the purse‐string suture is indispensable for nipple correction as it supports the nipple and maintains the shape. In cases where the sutures are not tight enough or are removed in advance, the nipple may collapse due to inadequate support. However, the sutures are usually placed under the deep layer of the nipple. Without a clear and direct sight of the sutures, the removal process is often conducted based on the experience of doctors, which increases the difficulty and the risk of residual knots. The remaining knots could act as foreign bodies that trigger inflammation and subsequently hinder postoperative recovery. Above all, the suture knots are often tied loosely, which poses a risk of insufficient purse‐string stability. To compensate for the loose knot and enhance therapeutic efficacy, the modified surgical technique has been innovatively experimented with in clinical practice, centered on improvements to the circular purse‐string suturing technique. The modified circular purse‐string suture begins at the base of the nipple on the side of the incision adjacent to the areola, proceeding with a subcutaneous circular suture. After reaching the opposite side of the incision, the suture thread is brought through the skin and tied to a 1 × 0.8 cm gauze strip, which is externally fixed to the areola on the opposite side of the incision. Subsequently, the suture re‐enters the cavity through the skin to complete the second half of the subcutaneous circular purse‐string suture fixation.

In summary, fistula incision and debridement combined with modified nipple correction not only suggests optimal tension in the sutures but also may be associated with the time of suture removal, thus technically enhancing the reconstruction effect. Moreover, the sutures can be easily removed thanks to the external gauze ball, resulting in a lower need for complex maneuvers deep within the surgical cavity. This modification reduces postoperative discomfort for patients and potentially allows for smaller surgical incisions. Still, the small sample size is a major limitation of this study. Therefore, the new procedure appears feasible and shows preliminarily encouraging outcomes. Compared with historical data, there is a potential trend toward clinical benefit. However, well‐designed randomized trials are needed to define its true value in the future.

## Author Contributions

Conception and design of the study: Bing Wang and Meina Ye. Acquisition and analysis of data: Bing Wang and Yue Zhou. Writing of the manuscript: Bing Wang and Jiachen Xu. Critical revision of the manuscript for intellectual content: Meina Ye.

## Funding

This work was supported by the 2024 Youth Academic Salon Project on Clinically Dominant Diseases by the Chinese Society of Traditional Chinese Medicine (No. 2024‐QNXSSL‐06), The Shanghai Shenkang Hospital Development Center’s Second Three‐Year Action Plan for Promoting Clinical Skills and Innovation in Municipal Hospitals (2020–2022)—Major Clinical Research Project (No. SHDC2020CR2051B), and The Major Difficult and Complicated Diseases Collaborative Project of Traditional Chinese and Western Medicine by the National Administration of Traditional Chinese Medicine: Plasma Cell Mastitis (ZDYN‐2024‐A‐019).

## Ethics Statement

This study was performed in line with the principles of the Declaration of Helsinki. Approval was granted by the Ethics Committee of Longhua Hospital Affiliated to Shanghai University of Traditional Chinese Medicine.

## Consent

Informed consent was obtained from all individual participants included in the study.

## Conflicts of Interest

The authors declare no conflicts of interest.

## Data Availability

Research data are not shared.
